# A rigid gas permeable contact lens discovered embedded in the upper eyelid 7 years after trauma: A case report

**DOI:** 10.1016/j.ijscr.2022.107316

**Published:** 2022-06-16

**Authors:** Sho Ishikawa, Minami Chino, Kei Shinoda

**Affiliations:** Department of Ophthalmology, Saitama Medical University, Saitama, Japan

**Keywords:** Case report, Rigid gas permeable contact lens, Foreign body, Rheumatoid arthritis

## Abstract

**Introduction:**

Rigid gas permeable (RGP) contact lenses implanted in the upper eyelid may remain asymptomatic. It is important to evert the upper eyelid even in the absence of definite pain.

**Presentation of case:**

A 74-year-old woman with a history of rheumatoid arthritis visited a local clinic because of right eye discomfort that had persisted for 5 months. Eversion of the upper eyelid revealed an embedded foreign body, and she was referred to our hospital. A transparent and smooth-surfaced foreign body was found embedded at the center of the upper eyelid conjunctiva. Additionally, fatty tissues were found behind the foreign body. Computed tomography (CT) and magnetic resonance imaging (MRI) revealed a foreign body rich in water at the same site. The foreign body was a spherical object with a diameter of 9 mm reaching as deep as the tarsus. On removal, the foreign body was identified as an RGP contact lens. On further questioning, it was discovered that the patient had lost an RGP contact lens 7 years earlier. The symptoms disappeared after removal of the foreign body.

**Discussion:**

RGP contact lenses are not detected on MRI scans, but cysts around the lenses are detected, which may result in multiple detections. CT cannot differentiate a foreign body from granulation tissue. The foreign body itself or a reactive granuloma can be seen when the eyelid is everted.

**Conclusion:**

A contact lens embedded in the eyelid without symptoms is rare but can be detected via a detailed interview, visual examination, and diagnostic imaging.

## Highlights

• Rigid gas permeable (RGP) contact lenses can become embedded in the eyelid asymptomatically.
• Upper eyelid eversion and palpation are important to detect foreign bodies.
• CT could not differentiate RGP contact lenses from granulation tissue.
• RGP contact lenses themselves are not depicted on MRI scans.
• Swelling of surrounding tissues is shown by high-intensity signaling on MRI scans.

## 1 Introduction

Rigid gas permeable (RGP) contact lenses rarely migrate into the conjunctival sac and become lost. Green et al. [1] reported the first case of migration of a contact lens into the conjunctival sac in 1963. Subsequently, several additional cases of spontaneous migration [2] and migration caused by trauma [4], [5], [6], [7] have been reported. The reported sites of migration include the upper fornix, tarsus, preseptal space, and orbit [2]. Although most cases remain asymptomatic, migrated contact lenses are sometimes discovered after the onset of symptoms such as discomfort, eye discharge, eyelid swelling, and ptosis [2], [3].

Here we report a case of a patient with an embedded RGP contact lens who had blurred symptoms but was unaware of the presence of a foreign body due to a history of a corneal ulcer. This work has been reported in accordance with the SCARE criteria [8].

## 2 Case report

A 74-year-old woman developed discomfort in the right eye and visited the local clinic that had prescribed her RGP contact lenses. However, no abnormalities were detected. The symptoms persisted, and she visited another eye clinic, where a foreign body was found embedded in the right upper eyelid. She was then referred to our hospital.

**Fig. 1:**
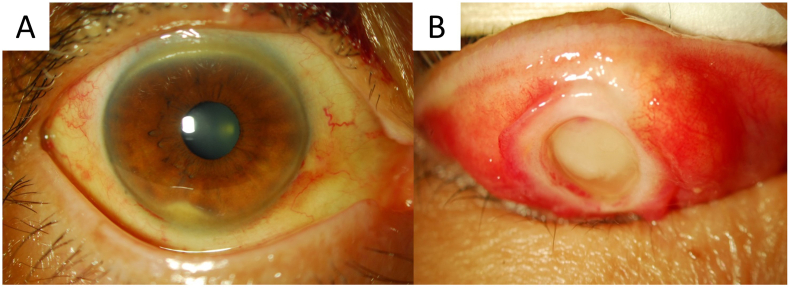
Photographs of the right eye taken at the initial visit. A. A photograph of the cornea of the right eye. The corneal stroma is opacified around the lower segment. B. Eversion of the right upper eyelid. A transparent foreign body and an approximately 11-mm mass covered with granulation tissue can be seen at the center of the eyelid. The foreign body at the center is transparent and smooth-surfaced and approximately 6 mm is exposed. There is fatty tissue beneath the foreign body. The foreign body was later confirmed to be a rigid gas permeable contact lens.

At the time of the patient's initial visit to our hospital, she had been experiencing symptoms for 5 months. She had a history of rheumatoid arthritis and had been receiving oral treatment with penicillamine, salazosulfapyridine, and loxoprofen sodium for 13 years. She had been treated for a peripheral corneal ulcer related to rheumatoid arthritis 10 years previously in the right eye. The Simplified Disease Activity Index and Clinical Disease Activity Index, which are used to assess the activity of rheumatoid arthritis, were 13.8 and 12.5, respectively, indicating that she had moderately active disease. The patient's daughter also had rheumatoid arthritis. The patient's corrected visual acuity was 0.7 in each eye, and the intraocular pressure was 10 mmHg and 12 mmHg in the right and left eyes, respectively. No abnormalities were observed in the eye position or movement. Although neither eyelid swelling nor ptosis was observed, an elastic soft mass was palpated in the right upper eyelid skin. The corneal stroma was opacified around the inferior corneoscleral limbus (Fig. 1). Although hyperemia was detected in the upper segment of the bulbar conjunctiva, fluorescein staining showed no abnormalities of the cornea or conjunctiva. Mild cataracts were observed in both eyes, but there were no abnormal findings in the fundus. Eversion of the upper eyelid revealed a transparent foreign body and a mass approximately 11 mm in diameter covered with granulation tissue at the center of the eyelid. The foreign body was smooth-surfaced with approximately 6 mm exposed, and there was underlying fatty tissue (Fig. 1). The patient had been using RGP contact lenses for 20 years. When questioned, she reported that she had never lost a contact lens.

**Fig. 2:**
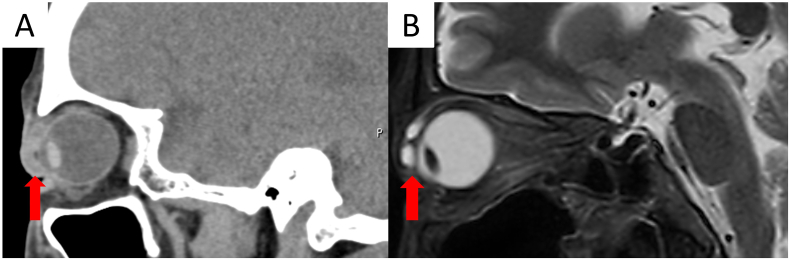
Imaging of the right eye. A. Orbital computed tomography showing a 17 × 15 × 8-mm mass that is slightly denser than the skin. B. Orbital T2-weighted magnetic resonance imaging showing two hyperintense and smooth-surfaced masses measuring 5 mm and 6 mm.

Computed tomography (CT) was performed to confirm the depth of the foreign body. It revealed tissue under the eyelid skin with a density different from that of the surrounding tissue. Because CT could not differentiate the foreign body from granulation tissue, magnetic resonance imaging (MRI) was performed. There were two hyperintense and smooth-surfaced masses showing as areas of low intensity in T1-weighted images and high intensity in T2-weighted images indicating that an object rich in water was present in the right upper eyelid (Fig. 2). We determined that the discomfort was caused by the foreign body and the surrounding granulation tissue.

**Fig. 3:**
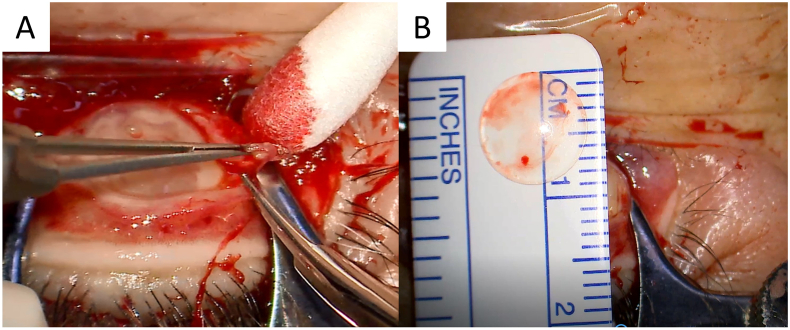
Photographs of the right eye taken during surgery. A. Resection of the surrounding granulation tissue revealing a circular object reaching as deep as the tarsus. B. The foreign body is an intact rigid gas permeable contact lens with a diameter of 9 mm.

Surgery was performed to remove the foreign body at Saitama Medical University (Sho Ishikawa). After resection of the overlying granulation tissue, the foreign body was removed. It was circular with a diameter of 9 mm and appeared to be an RGP contact lens. It was embedded with its convex surface facing toward the cornea. After removal of the contact lens, fatty tissue and the tarsus were identified beneath it (Fig. 3). Culture of the ulcer base, where the contact lens had been embedded, yielded no bacteria. When we asked about the loss of an RGP contact lens again after completing the surgery, the patient's family told us that she lost an RGP contact lens 7 years ago when she bumped her eye on a doorknob. After the trauma, she had purchased a new set of RGP contact lenses and had been using the replacement contact lenses for the past 7 years. Histological examination revealed that the granuloma around the contact lens contained mucosal tissue covered with squamous and pseudostratified columnar epithelium and granulation tissue under the epithelium. Two months after removal of the contact lens, the symptoms disappeared, and the defect in the upper eyelid gradually healed without complications.

## 3 Discussion

This was a rare case of an RGP contact lens embedded in the tarsus of the upper eyelid after trauma. It was only discovered after the onset of symptoms suggesting the presence of a foreign body.

The mechanism by which RGP contact lenses become embedded in the eyelid after trauma has not been determined. It is assumed that, when the eyes are rapidly closed by the blink reflex, the contact lens moves and adheres to the upper eyelid fornix and then gradually becomes covered with conjunctival epithelium, ultimately becoming embedded [4]. In this case, the patient had continued wearing RGP contact lenses after the loss of the contact lens. Thus, the lost contact lens might have become further embedded by the new contact lens moving into the conjunctival sac when the eyes were closed, due to Bell's phenomenon, and pressing on the lost contact lens. As the patient had been using oral analgesics for rheumatoid arthritis, this may have prevented her from feeling pain. Consequently, the contact lens may have become further embedded.

A literature search revealed five reported cases of an RGP contact lens becoming embedded in the eyelid following trauma [3], [4], [5], [6], [7]. Only one of the five reports described formation of granulation tissue and a capsule around the contact lens [3]. In the other reports, the contact lens did not cause a foreign-body reaction. A possible reason for this is that migration of a contact lens due to trauma is less likely to cause tissue disruption than spontaneous embedding of a contact lens [4]. In our case, the contact lens might have caused a foreign-body reaction because of the granulomatous changes around the contact lens. There are two possible explanations for this. First, the patient had continued wearing RGP contact lenses after the loss of the first contact lens. Thus, we assume that granulomatous changes were caused by friction between the two contact lenses or between the embedded RGP contact lens and the conjunctival epithelium covering it. Second, the patient had a history of rheumatoid arthritis. Patients with highly active rheumatoid arthritis have been reported to have elevated proinflammatory cytokine levels on the ocular surface [9], [10]. The patient had moderately active disease. In addition, she had stromal opacification in the lower corneoscleral limbus at the site of the previous corneal ulcer, indicating healed inflammation. She may have had a propensity to develop inflammation of the eyes. When an RGP contact lens is incompletely embedded, a closed space is created between the conjunctiva and the RGP contact lens. Bacterial infection in this space may cause granulation. In this patient, there was no infiltrate or abscess around the embedded contact lens, and the culture detected no bacteria. Based on these findings, infection is unlikely.

MRI revealed multiple nodular lesions with a high-intensity signal on T2-weighted images. MRI performed at the time of migration of an RGP contact lens detects proteinaceous cysts around the lens [11] and shows swelling of the surrounding tissues [3] instead of the lens itself. This probably explains why multiple hyperintense lesions were detected.

## 4 Conclusions

This report describes a case of an RGP contact lens becoming embedded in the upper eyelid after trauma. Although the patient remained asymptomatic for 7 years, the development of granulation eventually caused symptoms, which led to the discovery of the embedded lens. Although RGP contact lenses generally do not cause foreign-body reactions, our patient may have developed a foreign-body reaction because of her rheumatoid arthritis. Moreover, the analgesics that she was taking for treating rheumatoid arthritis may have masked the symptoms of the embedded contact lens. In patients who report the loss of an RGP contact lens, even those without symptoms, it is important to evert the upper eyelid of the eye to check for the presence of the missing lens.

## Ethical approval

Exempt from ethical approval.

Saitama Medical University Hospital.

## Funding

This research did not receive any specific grant from funding agencies in the public, commercial, or not-for-profit sectors.

## Author contribution

Sho Ishikawa: Writing Original Draft, Review and editing.

Minami Chino: Review and Editing.

Kei Shinoda: Conceptualization and Supervision.

## Guarantor

Sho Ishikawa.

Kei Shinoda.

## Registration of research studies

None.

## Consent

Written informed consent was obtained from the patient for publication of this case report and accompanying images. A copy of the written consent is available for review by the Editor-in-Chief of this journal on request.

## Provenance and peer-reviewed

Not commissioned, externally peer-reviewed.

## Declaration of competing interest

The authors declare that they have no conflict of interest.
